# Long-Term Steady-State Dry Boreal Forest in the Face of Disturbance

**DOI:** 10.1007/s10021-019-00455-w

**Published:** 2019-10-30

**Authors:** Christopher Carcaillet, Mireille Desponts, Vincent Robin, Yves Bergeron

**Affiliations:** 1 Laboratory for Ecology of Natural and Anthropised Hydrosystems (UMR 5023 CNRS ENTPE UCBL), Université Lyon 1, 69622 Villeurbanne, France; 2 Paris Sciences and Lettres Université (PSL), École Pratique des Hautes Études (EPHE), Paris, France; 3 Ministère des Forêts, de la Faune et des Parcs, Gouvernement du Québec, Direction de la recherche forestière, 2700 rue Einstein, Québec, Québec G1P 3W8 Canada; 4 Interdisciplinary Laboratory for Continental Environments (LIEC), University of Lorraine, CNRS, Campus Bridoux, Rue du Général Delestraint, 57070 Metz, France; 5 Institut de recherche sur les forêts, Université du Québec en Abitibi-Témiscamingue, 445 boulevard de l’Université, Rouyn-Noranda, Québec J9X 5E4 Canada

**Keywords:** Perturbation, Resilience, Resistance, Fire, Paleoecology, Macrofossil, Quebec

## Abstract

We used bioproxies from paleosoils buried within two aeolian dunes to test hypotheses concerning the origin of dry sandy boreal forests in Canada. These forests are dominated today by *Pinus banksiana* Lamb. One hypothesis is that too frequent Holocene stand-replacing fires would have transformed the original vegetation through extirpation of susceptible species to fire in water stress habitat. Alternatively, the ecosystem would have not changed since the dunes stabilized enough to support forest establishment. The vegetation composition and richness were determined by identification of charcoal and macroremains and radiocarbon dating for the chronology. Both sites revealed a similar history covering 6400 years. Half of the charcoal layers were less than 2500 years old in both sites, indicating that they had been subjected to the same fire history. Data indicated a stable plant composition and richness, although the percentage of *Pinus* decreased slightly over 4000 years (decreasing rate 1% per century). The fungus *Cenococcum geophilum* was consistently present, with a stochastic abundance. The vegetation grew under natural fire conditions and soil dryness since 6000 years. The ecosystem was probably not stressed by late-Holocene fires or climate changes, as the multi-millennial steady state reveals a resistant and resilient ecosystem.

**Electronic supplementary material:**

The online version of this article (10.1007/s10021-019-00455-w) contains supplementary material, which is available to authorized users.

## Introduction

**Figure 1:**
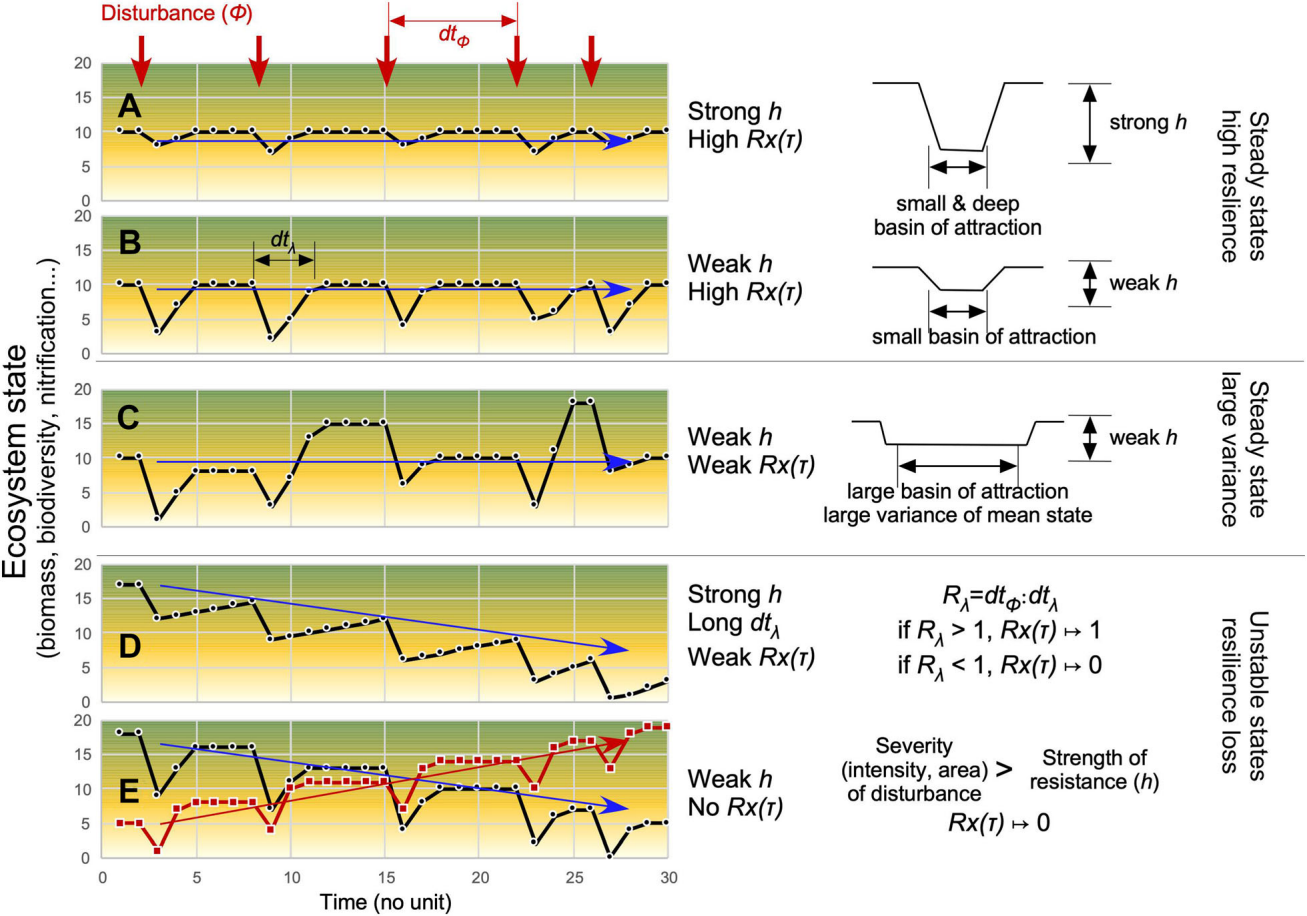
Conceptual diagram of the cumulative effects of a certain disturbance (red arrows, *φ*), on the temporal stability of a regularly disturbed system; in this workout, the type of disturbance (for example, fire), its intensity (energy given off; Johnson 1992) and its frequency (event numbers per time unit) are constants. (A) Over time, the resistance of the ecosystem (*h*) is strong enough to secure the recovery and thus the full temporal autocorrelation of ecosystem state [*Rx*(*τ*) = 1] measured by comparison between state values before and after the disturbance (biomass, biodiversity, interaction networks, biogeochemistry, etc.); in this case, the resilience (*λ*) is maximal and the system is long-term steady. (B) The resistance (*h*) is weak, but the resilience is strong because the recovery rate is strong and rapid enough thus allowing a full autocorrelation. (C) The resistance (*h*) and the autocorrelation [*Rx*(*τ*) < 1] are weak, but the basin of attraction is large enough to guarantee the long-term stability of the system (metastable); the high variance of the system is due to hazards of disturbance parameters (season, severity, area) or of post-recovery features (diaspore abundances, weather, interactions with other disturbances, etc.), which control processes (biogeochemistry, productivity) and biodiversity (species traits, demography, biotic interactions, etc.); the large basin of attraction (variance) can be a strength to respond to stress and disturbances and display to high eco-diversity, including internal state of maturation within the large basin of attraction (Müller and others 2016). (D) The ecosystem is unstable because the resilience ratio (*R*_*λ*_) between the disturbance interval (d*t*_*Φ*_) and the recovery time (d*t*_*λ*_) does not guarantee anymore the conservation of the ecosystem functionalities and thus of states [$Rx(\tau ) \mapsto 0$]. Over time, the system changes progressively of attraction basin following staircase dynamics. (E, black) The ecosystem is unstable, and its value decreases because of the strength of the disturbance (intensity, extent) largely above the resistance or recovery potential of the system (resilience disequilibrium) affecting its functionalities; (E, red) the instability of the system outcomes from higher value after each disturbance by increasing stock or availability of nutrients resulting a progressive augmentation of ecosystem state values; this trajectory was well conceptualized by McLauchlan and others (2014). Both staircase dynamics (black or red) result a change in attraction basin following a monotonic multi-secular trajectory. The terminology and definitions of resilience terms are provided in Supporting Information (Text S1, Figure S1).

In boreal regions, the long-term compositional origin of forests on dry soils has important conservation implications (for example, regarding biodiversity, carbon, soil erosion) because of industrial tree exploitation and other global changes that can threaten these boreal ‘snow’ forests (Moen and others 2014; Gauthier and others 2015). Boreal forests are generally fire prone. If we can determine the origin of such fire-prone ecosystems, that is, progressive transformation *versus* initial spontaneous emergence, we may also understand the ecosystem linkage between community and disturbance and, in particular, whether a change in disturbance regime could result in a transformation of community or whether a steady state would be maintained for a long time whatever the disturbance regimes. These hypotheses question the resilience of the system. Two long-term scenarios can be considered (Figure 1). First, we hypothesized that the initial postglacial ecosystem was not appropriate for stand-replacing fires, and that frequent fires have progressively altered the ecosystem by limiting or increasing its functionalities (Figure 1D, E), for instance, the plant recruitment due to effects on seed bed properties or on seed productivity of trees (too short fire interval according to tree reproductive maturity, for example, Viglas and others 2013) or by progressive decrease in biogeochemical mechanisms (McLauchlan and others 2014), promoting the most fire-adaptable species over species less resilient or resistant. The unknown initial system would have progressively or abruptly moved to an alternative steady state or basin of attraction (Scheffer and others 2001), based on the interaction between the disturbance regime and other environmental stressors that promote transformation of ecosystems (McLauchlan and others 2014; Oliver and others 2015). Second, we hypothesized that the ecosystem was functionally resilient to frequent fires as soon as the habitat was colonized after deglaciation without intermediate states (Figure 1A–C), whatever climatic changes and soil maturation processes that drive the main natural chronic or rapid environmental changes in boreal habitats.

In North America, snow forests on dry soils are dominated by *Pinus banksiana* Lamb., from the Northwest Territories and Alberta in the west, to Nova Scotia in the east, Wisconsin–Michigan in the south and the Canadian forest–tundra transition in the north. Fire pattern could have controlled some long-term variation in vegetation composition under climatic change (Genries and others 2012; Lynch and others 2014), and modern populations might have arisen from the fragmentation of populations at the landscape scale since the middle Holocene approximately 6000 years ago (Payette and others 2017). However, it is unclear whether the modern ecosystem—*P. banksiana*, forests on dry soil—is the legacy of past processes (disturbances, climate) that have progressively reformed the initial system by rarefaction or extirpation of susceptible species, or whether the modern dominant tree species appeared immediately after deglaciation and have been sustained dominant until today in a steady state. Indeed, it has been shown that afforestation of pines might have occurred without lag soon after the deglaciation in northeastern North America (Blarquez and Aleman 2016). In the North American boreal shield, other species in this system, potentially susceptible to fire and soil dryness, include black spruce (*Picea mariana* Mill. BSP) and white spruce (*Picea glauca* (Moench) Voss.), respectively, balsam fir [*Abies balsamea* (L.) Mill.] and eastern white cedar (*Thuja occidentalis* L.) (for example, Visnadi and others 2019). Broadleaved boreal trees (for example, *Betula papyrifera* Marshall or *Populus tremuloides* Michx.) are not expected to abound on sandy soils because they require too much water.

**Figure 2:**
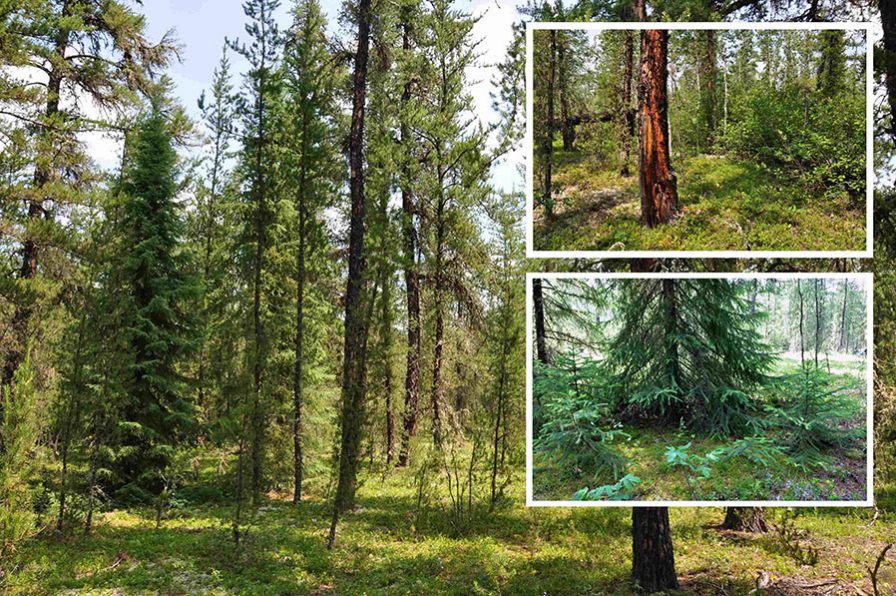
(left) Old-growing boreal forest (multi-aged) on dry sandy soils dominated by jack pine, *Pinus banksiana*, with intermingled understory of ground lichen and feathermoss, covered by *Vaccinium vitis*-*idaea* and presence of *Hudsonia tomentosa* on bareground soil surface; the tree community includes scattered *Betula papyrifera* and *Picea mariana* (left side of the picture) showing vegetative propagation by layering; (top right) detail on layering of *P. mariana*; (low right) fire scar on trunk of *Pinus banksiana*, and tall shrub of *Alnus viridis* photograph credits: Chris Carcaillet.

Ecosystems with dry sandy soil dominated by *P. banksiana* are currently sustained by frequent fires (for example, Lavoie and Sirois 1998; Le Goff and Sirois 2004; Tweiten and others 2015), and fire occurrences are sustained by high *P. banksiana* density (for example, Héon and others 2014), making these forests fire-prone ecosystems that are seemingly resilient (Day and others 2017; Hart and others 2018). The positive feedback results in intimate interactions between the fire and ecosystem processes. Indeed, *P. banksiana* presents response traits to the fire regime (Gauthier and others 1996; Radeloff and others 2004; de Groot and others 2004; Briand and others 2015) and controls the fire spread by effect traits (Fonda 2001). Furthermore, *P. banksiana* dominant ecosystems are also often associated with rocky outcrops, fluvioglacial deposits or aeolian dunes (Schmidt and Carmean 1988) covered by ground lichens (*Cladina* type), all of which are substrata that stimulate fire spread as a result of soil dryness and fuel quality. These ecosystems are generally nutrient-poor and dependent on mycorrhizal fungi that are stressed by ground lichens (Pacé and others 2019a, 2019b) either through allelopathy (Brown and Mikola 1974) or other soil biogeochemical processes (Sedia and Ehrenfeld 2006). Precisely, ground lichens reduce the nutrient availability, notably phosphorus and base cations and also release leachates that affect the pine seedling growth, whereas with moss cover on same substratum, the growth of pine seedling is stimulated by fungal development that improves nutrient uptake for plant through mycorrhizal association (Pacé and others 2019a, b). Further, with ground lichens, the diversity and abundance of mycorrhizal fungi are lower, probably explaining the depleted nutrition by plants (Pacé and others 2019a, b). These forests are often characterized by even-aged pine canopies, with post-fire tree seedling density and understory composition linked to burn rate or fire severity. When the interval between successive stand-replacing fires is short, young stands dominated by serotinous *P. banksiana* are susceptible to natural regeneration failure due to the time required to produce a seed supply deemed adequate to regenerate a stand, thereby leading to a decline in productive forest cover (Pacé and others 2019b). Very severe fire can burn cones and limit regeneration (Pinno and others 2013), whereas low fire severity on the ground may decrease good seed beds as seedlings need an access to mineral soil to survive drought (Greene and others 2007). Burning of all organic matter by very severe fire can, however, impair stand productivity. However, multi-aged *P. banksiana* communities with scattered *Picea mariana* and intermingled cover of ground lichen and bryophytes (Figure 2) can be found when fire intervals are long or fire intensity is low (Smirnova and others 2008). In such weakly disturbed forests with ground mosses (Figure 2), the regeneration and growth rate of *P. banksiana* is improved, probably due to a more intense and diverse mycorrhizal process facilitating pine nutrition (Pacé and others 2019a, b).

Here, we thus test the hypothesis that the modern *P. banksiana* dry soil ecosystem has arisen from initial mixed communities present in the study area since the deglaciation (*Picea* × *Pinus* × *Abies* × *Thuja* × *Betula* × *Populus*; Liu 1990; Richard 1995; Carcaillet and others 2001), from which the species most susceptible to dryness (*Picea mariana, P. glauca*, *Abies balsamea*, *Thuja occidentalis*, *Pinus strobus* (L.), *Betula papyrifera*) would have been progressively extirpated as a cumulative result of fires on biogeochemical processes, plant traits or trophic networks, whatever their intensity or their severity (Figure 1D, E). Because *P. banksiana* promote fire, the fire frequency would have increased over time after the inception of the forest (positive feedbacks). Alternatively, *P. banksiana* would have been the dominant species as soon as the dune ecosystem was established during the postglacial period, and the fire regime has remained stable as a result of the pine–fire interactions, thus offsetting the role of climate changes on the plant composition or fire regime (Figure 1A–C). Evidences for the first hypothesis abound in the literature where changes in vegetation and fires occurred during the Holocene progressively (for example, Carcaillet and others 2010; Couillard and others 2018) or abruptly (for example, Jasinski and Payette 2005) moving ecosystems from steady states to another, eventually with intermediate states.

The long-term origin of a forest can be assessed using paleoecological methods. Here, to reach a high spatially accurate analysis of past vegetation and fire, in the absence of peat hollows or very small ponds (few 0.01–0.1 ha) within dunes, we analyzed stratified paleosoils buried in thick dunes (Matthews and Seppälä 2014). This approach is highly spatially accurate although records are very discreet in time and not continuous. Botanical identification of soil charcoal combined with radiocarbon measurements allowed reconstruction of past burned woody vegetation (for example, Robin and others 2013), and subfossil plant macroremains (Birks and Birks 2000) were used to reconstruct past stands of unburnt vegetation (Supporting information S2 on taphonomy of soil/dune charcoal, plant and fungus macroremains).

## Materials and Methods

### Study Area

**Figure 3:**
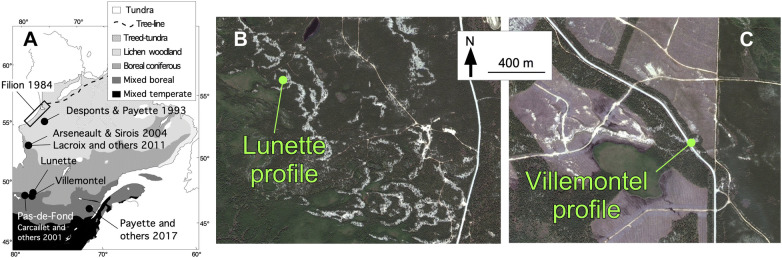
(**A**) Vegetation map of eastern Canada, with indications of the present study sites (Lunette, Villemontel) and main studies or sites mentioned in the text, which contribute to illustrate the Holocene history of *Pinus banksiana* forests or fire history in context of dry soils ecosystems. (**B**) Aerial view of the dune field of Lunette, showing the undisturbed parabolic shape of dunes (light-gray) covered by *Pinus banksiana* forests. (**C**) At Villemontel, the forest has been cut and wood harvested probably in 2010, excepted along the main road (Image Landsat/Copernicus 2011; ©DigitalGlobe 2019).

The study was conducted in Abitibi, western Quebec (Canada; Figure 3A), about 300 km south of James Bay, near the Ontario border in the ‘northern clay belt.’ This natural region, covering a large area in Ontario and Quebec, comprises a wide plain of the central Canadian shield where main wet and mesic soils overly clay deposited by the proglacial Lake Ojibway (Veillette 1994). The proglacial lake drained abruptly when the residual ice sheet collapsed, allowing a northward outflow to the Hudson Bay, about 8200 calibrated years before present, hereafter ‘cal BP’ (Barber and others 1999). Theoretically, dunes could thus not have accumulated before about 8200 cal BP. The Abitibi topography is a rolling landscape covered by clayed soils intersected by fluvioglacial deposits (eskers, moraines, sand cover). The Abitibi plains have been wind eroded for 8000 years and have developed parabolic aeolian dunes on clay deposits of the proglacial Lake Ojibway (Figure 3B). During relatively stable periods, woody vegetation grew on these dunes, producing soils (phase of biostasy). Fires then burned the woody vegetation, resulting in charred particles of wood, cones, needles, seeds, etc., which were later covered by sandy deposits during new active aeolian periods (rhexistasy). Such processes have produced paleosoils stratified in thick sandy deposits. They have been well studied in North America and Europe (for example, Filion 1984; Seppälä 1995). Several fires can occur before the burial of paleosoils containing charcoal from these accumulated fires. This type of sandy deposits represents an important source of paleoecological information in dry boreal areas where or when lake sediments or peat is rare (for example, Bélanger and others 2014; Matthews and Seppälä 2014).

### Study sites

Two aeolian dunes were excavated manually with shovels close to the small locality of Villemontel, located (site hereafter named ‘Villemontel’; Figure 3C, 48.6977°N–78.3630°W) and the nearby Lake Lunette (hereafter ‘Lunette’; Figure 3B, 48.7991°N–78.3886°W). These dunes are situated on the Lake Berry esker, which is about 150 km long. This esker has been transgressed and eroded by the proglacial Lake Ojibway, resulting in sandy paleobeaches (Allard 1974). This sand occurs at the origin of the parabolic dunes (Figure 3B), notably the Villemontel dune, which are on the summit of the Lake Berry esker. The Lac Lunette dune corresponds to a wide forest area interrupted by dunes about 1.2 km west of an esker (Figure 3B). Both dunes are about 3 m thick. At both sites, at the sampling time, the modern forests were dominated by *P. banksiana approximately* 70 years old in Villemontel and 50 years old in Lunette (Table 1), with scattered *P. mariana*, *Betula papyrifera* and *Populus tremuloides*. The canopies were at about 20 m at Villemontel with a tree density of 25–40% and about 10 m in Lunette with a density greater than 80% (Table 1). The understory is dominated by *Kalmia angustifolia* L., with *Vaccinium* L. spp., *Hudsonia tomentosa* Nutt. and other forbs and herbs, and lichens (*Cladonia* P. Brown sp.) and mosses (*Polytrichum piliferum* Hedw.). Both dunes are situated in a transition zone between mixed (southern) and closed-crown (northern) coniferous boreal forests. In 2010, the forest was partly clear-cut on the site of Villemontel (Figure 3C).

**Table 1:** Provincial (Québec) forest inventory data for both study sites

Date of satellite image	Lunette 48.7991°N–78.3886°W (333 m)			Villemontel 48.6977°N–78.3630°W (339 m)		
	Stand age	Tree density	Tree height	Stand age (years)	Tree density (years)	Tree height (m)
1972	Burned	–	–	40	60–80	12–17
1983	10 years	40–60%	4–7 m	50	40–60	12–17
2002	30 years	60–80%	7–12 m	70	60–80	17–22
2007	50 years	> 80%	7–12 m	70	25–40	17–22

Source: http://mffp.gouv.qc.ca/les-forets/inventaire-ecoforestier/. All the data are interpretations estimated from the satellite images.

### Dune Sampling and Chronology

The profiles were dug until the base was reached at Villemontel, and until no traces of pedogenesis activity were observed at Lunette. The clay deposit was never met both in Lunette and Villemontel. The dune profiles were cleared for a width of 1 m. Both sedimentary sequences comprised sandy material interrupted by charred organic accumulations. Each organic layer corresponded to a past stabilized soil that had burned at least once. The granulometry was measured in laboratory along the profiles.

Eight charred layers were dated at Villemontel and five at Lunette (Table 2). They were dated by ^14^C measurements at the GEOTOP center (Université du Québec à Montréal, Canada; measurements labeled UQ) and at the Centre d’Études Nordiques (Université Laval, Canada; UL). A bulk of charcoal or plant macroremains (Table 2) was preferred to a single remain in order to reduce the dating risk of older material reworked by blowing to the dune. The ^14^C measurements were calibrated with CALIB 7.0.4 (Stuiver and Reimer 1993) using the IntCal13 dataset (Reimer and others 2013). The CALIB tool *sum probabilities* were used with all the ^14^C measurements to compute cumulative probability distribution per site with two sigma ranges. After calibration, the radiocarbon dates were used to establish age–depth models by exponential smoothing, one per dune profile (cf. in Carcaillet and others 2006). The age–depth models were then used to simulate the ages of undated burned paleosoils. One measurement in each radiocarbon series appeared to be too old compared with the other data: 3316 ± 85 BP (UQ-2117) at 175 cm at Villemontel and 1870 ± 80 BP (UL-1268) at 49 cm at Lunette. These probably old measurements were thus not used for the establishment of the age–depth models (cf. Carcaillet and others 2006). The age–depth distributions clearly indicated an increase in the accumulation rate of sediment in the upper layers of both dune profiles, which was associated with an increase in the number of charred layers. The intervals between the charred layers were regular in terms of thickness rather than in terms of duration.

**Table 2:** Radiocarbon dating of charcoal layers from the Villemontel and Lunette Dunes in Abitibi, West Quebec, Canada

Depth (cm)	Lab. Code	^14^C years BP	Range of calibrated years BP (probability)	Dated material
Lunette				
49	UL-1268	1870 ± 80	1613–1989 BP (1.00)	Plant macroremains
89	UL-1629	1760 ± 100	1415–1464 BP (0.04)	Wood charcoal
			1477–1506 BP (0.01)	
			1513–1896 BP (0.95)	
173	UL-1630	2520 ± 70	2379–2415 BP (0.05)	Wood charcoal
			2420–2751 BP (0.95)	
193	UL-1631	3130 ± 80	3144–3512 BP (0.98)	Wood charcoal
			3527–3558 BP (0.02)	
209	UQ-2114	4314 ± 90	4613–4766 BP (0.15)	Wood charcoal
			4783–5079 BP (0.74)	
			5104–5136 BP (0.02)	
			5163–5281 BP (0.09)	
Villemontel				
17	UQ-2121	785 ± 52	655–796 BP (1.00)	Wood charcoal
39	UQ-2122	1144 ± 68	930–1186 BP (0.95)	Wood charcoal
			1205–1240 BP (0.05)	
127	UQ-2125	1933 ± 59	1722–1998 BP (1.00)	Wood charcoal
150	UQ-2126	2125 ± 45	1989–2184 BP (0.84)	Wood charcoal
			2233–2305 BP (0.16)	
175	UQ-2127	3316 ± 85	3368–3726 BP (0.97)	Wood charcoal
			3750–3762 BP (0.01)	
			3793–3821 BP (0.02)	
187	UL-1632	3270 ± 100	3246–3309 BP (0.04)	Wood charcoal
			3318–3724 BP (0.95)	
			3795–3818 BP (0.01)	
203	UQ-2116	3319 ± 88	3368–3726 BP (0.97)	Wood charcoal
			3750–3762 BP (0.01)	
			3793–3821 BP (0.02)	
222	UQ-2113	5525 ± 72	6168–6454 BP (1.00)	Wood charcoal

UQ corresponds to measurements made at the GEOTOP laboratory (Université du Québec à Montréal, Québec, Canada) and UL to the Centre d’Études Nordiques laboratory (Université Laval, Québec, Canada).

### Charcoal Identification

About half a litter of charred layer was sampled. Charcoal was extracted from sediments by flotation, and charcoal volumes per layer were measured by displacement of water in a beaker. The measured volume was expressed according to the volume of sampled sediment also measured by water displacement (vol_CHAR_/vol_SEDIMENT_ = %). The charred particles were very fragmented and looked like a black powder. Where possible, 30 fragments per layer were randomly selected for identification by picking them from the sieve (mesh size >350 µm). The identifications were based on wood anatomical structures using an incident light microscope (× 200, × 500, × 1000) and were compared with descriptions of wood in atlases (for example, Jacquiot 1955; Schweingruber 1990) or with wood charcoal in personal reference collections (CC, VR). *Larix* and *Picea* could not be distinguished because of their close anatomy (Marguerie and others 2000), resulting in the lumped charcoal taxon *Larix*/*Picea*. Three pine species can theoretically be observed in the area: *P. banksiana*, *Pinus strobus* and *Pinus resinosa* Aiton. They all present different anatomical structures belonging to three anatomical groups of pine, which secures their identifications at the species level (Jacquiot 1955). Particular attention was devoted to the size and pattern of medulla cells and to the abundance of vessels (transversal section) of Ericaceae wood charcoal for identification to species level wherever possible (Talon and Carcaillet 2004) and to wood porosity for identification of *Betula* tree *versus Betula* shrub (Hellberg and Carcaillet 2003). Identifications of plant in the collection respect the flora of Québec (Marie-Victorin and others 1995).

The abundance of charcoal taxa per assemblage was expressed as a frequency (%), that is, the number of fragments of a given taxon in relation to the total number of identified fragments within a collection of fragments. The occurrence rate was the ratio of the number of assemblages containing a given taxon to the total number of assemblages, whatever the relative abundance of the taxon in each assemblage. The occurrence rate allowed the ubiquity in the environment of the burned taxon to be characterized. For example, a low-abundant species that is always present in the environment (dispersed distribution) has a high rate of occurrence, while a locally abundant species (high local frequency) that is infrequent at the landscape level (having a clustered distribution) has a low occurrence rate. The taxon abundance and occurrence rates are two complementary proxies of fuel mass structure at the landscape level (Carcaillet 2017). Ideally, the burned biomass could be assessed, but consumption biomass and the charcoal production rate should first be calibrated (Fréjaville and others 2013); unfortunately, such data do not exist for these species.

### Plant Macroremain and Fungus Identification

One 600 cm^3^ sample was retrieved from each layer of charred organic material in the paleosoils for plant macrofossil analysis. Samples were soaked during 45 min at 90°C in a 10% KOH solution. The bulk solution was sieved at 150 µm. Identifications were made under a dissecting microscope. The results are expressed as number of pieces. For identification, the remains were compared with plant fragments in reference collections at the Department of Geography of the Université de Montréal, Québec, Canada. This collection of plant fragment was established by Mr. Alayn Larouche, based on identification respecting the flora of Québec (Marie-Victorin and others 1995). A particular attention was given to *Cenococcum geophilum*, an ectomycorrhizal fungus often counted in paleoecological studies based on macroremains (for example, Scott and others 2010; Lacroix and others 2011). All their sclerotes were counted. This fungus *C. geophilum* characterizes stress communities, notably as a result of chronic drought (Pigott 1982; Coleman and others 1989; Hasselquist and others 2005). Macroremains must be interpreted in the presence, not in the absence (Supp. Info. S2).

## Results

### Stratigraphy and Sedimentology

In total, 17 charred layers were observed at Villemontel and nine at Lunette. At Villemontel, all sands above the deepest layer (no. 17; 221 cm below the surface) were from aeolian transportation, with a modal size distribution of 177–250 µm, that is, fine sands. Below layer no. 17, there were coarser sands, heterogeneous in size and poorly sorted, including gravel-type particles larger than 20 mm and a rolled pebble layer (5–50 mm), which could be the summit of a paleobeach. At Lunette, all sands were of aeolian origin, with a modal distribution of fine sands of 177–250 µm. The aeolian dune was excavated more than 1 m below the deepest layer (no. 9; 208 cm) without any traces of pedogenesis being found.

### Wood Charcoal Identifications

**Figure 4:**
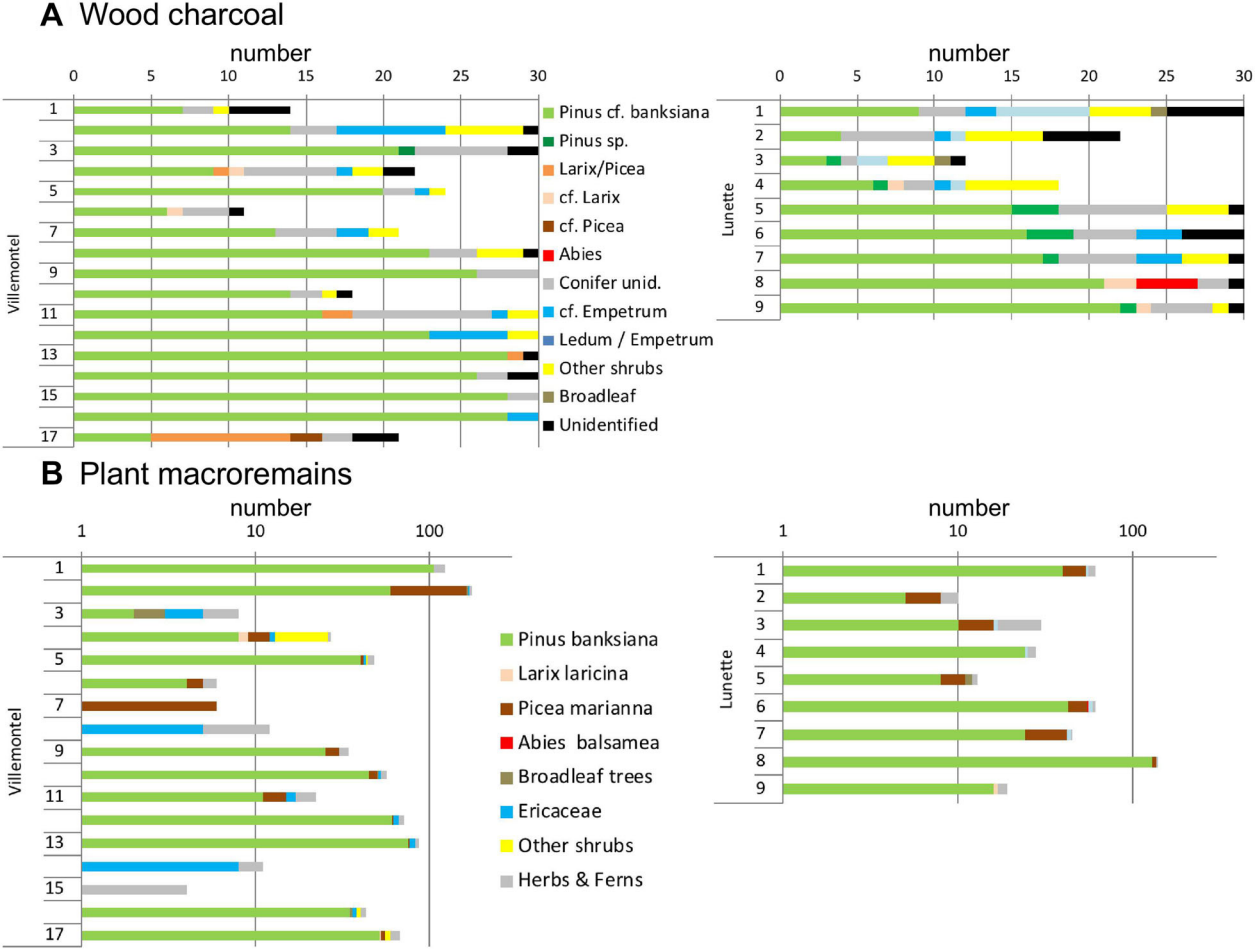
Wood charcoal (number) (A) and plant macroremains (B) (number per cm, log scale) plotted against depth, from the top layer (no. 1) to the deepest (no. 17 at Villemontel [left] and no. 9 at Lunette [right]). Layers are stratified; each layer corresponds to a paleosoil containing charcoal fragments from one or several fires. Details in Table S1 and S2.

**Figure 5:**
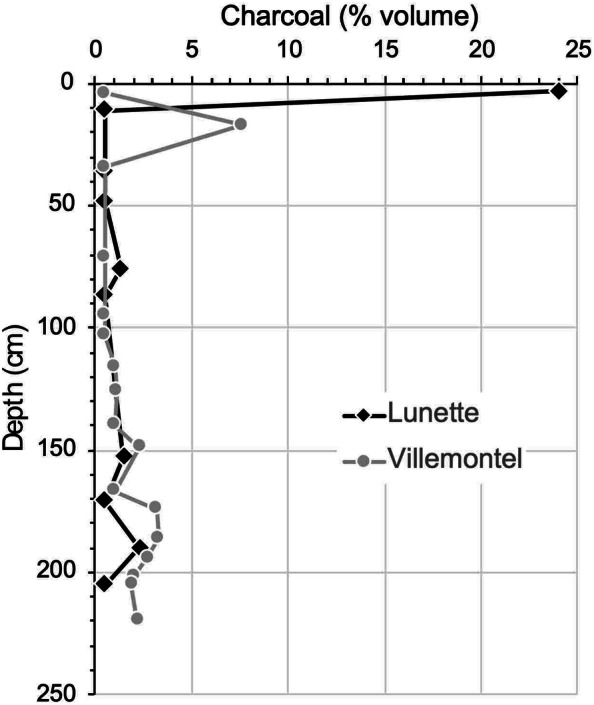
Charcoal concentration profile according to depth below the modern dune soil.

In general, the charcoal fragments were very small, often around 1 mm in size. In total, 232 and 431 fragments were identified at Lunette and Villemontel, respectively (Figure 4A). Large charcoal fragments were more abundant in the deepest layers explaining why more fragments were identified in these layers. Their measured volumes were generally higher in the lower-half layers (Figure 5), excepted in the topmost layer in Lunette (3 cm below the dune surface) and in the second topmost layer in Villemontel (17 cm below the surface). At Villemontel, if this 17-cm-depth layer is not considered, the volume is significantly lower in the top-half profile compared to the lower-half profile (Wilcoxon test, *p* = 0.0016). At Lunette, the difference is not significant even when the 3 cm layer was excluded.

In the upper layers, fragments were too small for identification. Of the 663 fragments, 8% were unidentified at Lunette and 4% at Villemontel, because of bad preservation of anatomical structures or because the fragments were glassy, an outcome of a burning process. At both sites, most charcoal fragments per assemblage corresponded with *P. banksiana*, which was present in all assemblages (Figure 4A, Tables S1 and S2). The deepest charcoal layer at Villemontel (no. 17) was the only layer where *P. banksiana* was not the most frequent taxon: *Larix*/*Picea* was the most frequent instead. The abundance of *P. banksiana* charcoal decreased with the decrease in depth. The other tree taxa recorded were cf. *Larix* (Villemontel no. 4 and 6), cf. *Picea* (Villemontel no. 17), *Larix*/*Picea* (Lunette no. 4, 8 and 9; Villemontel no. 4, 11, 13, and 17) and *Abies* (Lunette no. 8). Few dwarf shrubs were identified: cf. *Empetrum* and *Ledum*/*Empetrum*. Apart from samples with 100% *P. banksiana*, the occurrence rates of all other taxa were low (Figure 4A).

### Macroremain Identifications

*Pinus banksiana* was the species with the highest occurrence rate (85%): Only four layers of the 26 did not contain any *P. banksiana* needles, male or female cone fragments or seeds (Figure 4B). When present, *P. banksiana* showed the highest frequency of remains per assemblage, between 60 and 95%. The other main species (Table S1-S2), based on occurrence rate, were *P. mariana* (Villemontel 65%, Lunette 78%) and *Arctostaphylos uva*-*ursi* (Villemontel 65%, Lunette 67%). Secondary species were *Aralia hispida* (Villemontel 53%, Lunette 33%), *Hudsonia tomentosa* (Villemontel 29%, Lunette 78%) and *Carex* sp. (two-sided seed; Villemontel 71%, Lunette 11%). All other species showed stochastic occurrences: *Abies balsamea*, *Larix laricina*, *Diervilla lonicera*, *Vaccinium angustifolium*, *Vaccinium vitis*-*idaea*, *Prunus pensylvanicum*, *Viola* sp., *Comptonia peregrina*, *Carex* spp. (two types), cf. *Panicum*, *Scirpus* sp., *Equisetum* sp. and *Lycopodium tristachyum* (Table S1-S2).

### Ecosystem Trajectories, Plant Richness and Fires

**Figure 6:**
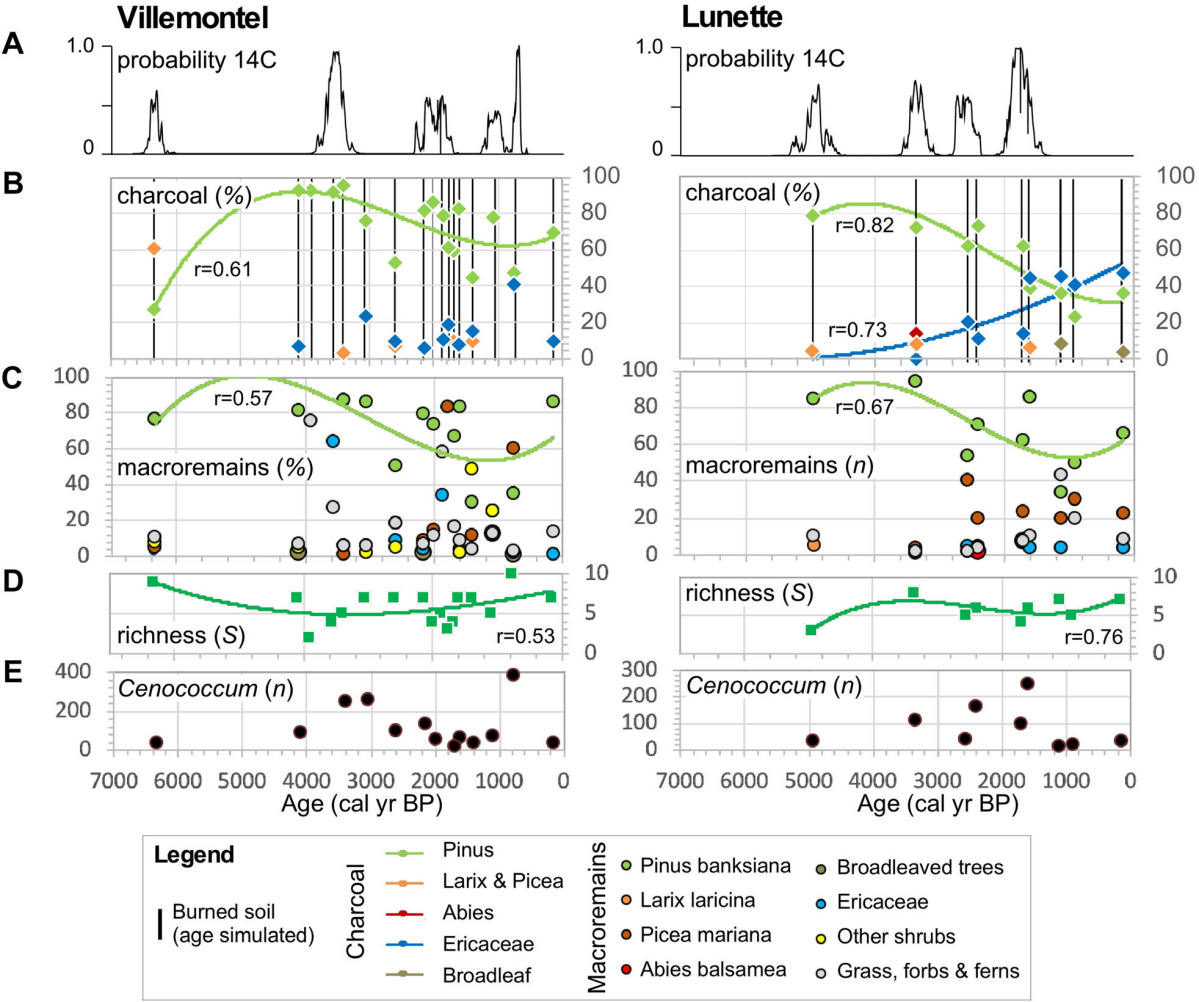
Ecosystem and biodiversity trajectories inferred from assemblages of charcoal and macroremains from paleosoils in western Quebec, Canada. (A) Probability sums of ^14^C dating. (B) Charcoal frequency (%) and temporal distribution modeled using polynomials (third order for *Pinus banksiana*; second order for Ericaceae); unidentified charcoal was used to calculate percentages; the vertical bars correspond to ages of burned paleosoils simulated based on age–depth models (cf. Carcaillet and others 2006); Pinus corresponds to identified charcoal of *P. banksiana* (major) and *Pinus* sp. (minor); Larix and Picea include all fragments identified as cf. *Larix*, cf. *Picea* and undifferentiated *Larix*/*Picea*. (C) Plant macroremain frequency (%) and temporal distribution of *P. banksiana* based on third-order polynomials. (D) Vascular plant richness based on pooled assemblages of charcoal and macroremains; time distribution based on third-order polynomials. (E) Sclerote numbers of *Cenococcum geophilum*, an ectomycorrhizal fungus.

The ecosystem trajectory could be illustrated by the different vegetation proxies, charcoal, plant macroremains and the sclerote abundance of *C. geophilum* (a fungus), and the richness pattern and fire history (Figure 6). The first layer of charcoal dated to *ca* 6350 cal BP at Villemontel and *ca* 5000 cal BP at Lunette (Figure 6A). The number of charred layers since 2500 cal BP at Villemontel (n = 9) and Lunette (n = 5) was of same order to the number recorded before (n = 7 and 4, respectively). However, the time intervals were shorter since 2500 cal. BP, suggesting an increase in aeolian activity associated with fires. Each charred layer may be a temporal mix of charcoal fragments from one or several fires and, eventually, a spatial mix if material was blown to the dune. This fact can explain anomalies of chronologies based on old ^14^C measurement in recent layers.

Both charcoal and macroremain assemblages clearly indicated that *P. banksiana* was the dominant species for at least 6350 years at Villemontel and 5000 years at Lunette. Interestingly, where the plant macroremains failed to reveal *P. banksiana* in four layers at Villemontel (Figure 6C), the charcoal showed that the *P. banksiana* was present with a high percentage of charred fragments (Figure 6B). At both sites, the percentage of *P. banksiana* decreased slightly during the last 4000 years, as illustrated by third-order polynomials: Villemontel *r* = 0.57; Lunette *r* = 0.67 (Figure 6B, C; values at 0% were not considered in the calculations because they correspond to absence). At Villemontel, no taxa compensated for this slight decrease in *P. banksiana*, while at Lunette, the assemblages indicated an increase in Ericaceae charcoal and in *P. mariana* macroremains. However, the results of assemblages differ each other, suggesting a stochastic abundance of Ericaceae and *Picea*. Similarly, at Villemontel, the first charcoal assemblage indicated an important frequency of *Larix*/*Picea*, a pattern not supported by the macroremain assemblage (Figure 6B, C). Generally speaking, the apparent difference between two proxies in one given layer (for example, Villemontel layer no. 8: mainly charcoal of Ericaceae *vs* macroremains of *P. banksiana*; Figure 6B) should not be understood as a true difference, but simply as a complementary effect of the two proxies, and therefore useful for better describing the diversity of the communities. In Villemontel no. 17, considering that there is no bias due to, for instance, the hazard of identified taxa (here charcoal) associated with low number of observations, the system may have been originally dominated by *Larix* or *Picea* and not by *Pinus*. Further, we also have to consider that the oldest assemblage contains plant remains that may have accumulated over a longer period of time than the later assemblages until first fire on the dune contributes to bury burned soils and associated remains. Finally, the spatial hazard of large woody debris on the ground must be considered as a source of representation bias of vegetation composition or biomass (Ohlson and Tryterud 2000; Bégin and Marguerie 2002; Ohlson and others 2013).

The richness curves were based only on the occurrence of vascular taxa. Both curves (third-order polynomials; *r* = 0.53 at Villemontel, *r* = 0.76 at Lunette) were steady without any significant temporal trend (Figure 6D). They depicted a mean richness ±SE (α-diversity) of 5.9 ± 0.7 taxa at Villemontel and 5.7 ±0.5 taxa at Lunette. These mean values were not distinguishable (Wilcoxon test, *p* = 0.82).

The ectomycorrhizal fungus *C. geophilum* was present in all layers at Lunette and 13 of the 17 layers at Villemontel (no data in layers no. 7, 8, 14 and 15). The distributions suggested important differences in abundances through time, may be stochastic (Figure 6E). However, no overall trends were evidenced at either site.

## Discussion

This multi-proxy study has revealed similar long-term histories at two sites, even though they were situated 7 km apart. The vegetation was dominated by *Pinus banksiana*, although its abundance decreased slightly over 4000 years. *Picea mariana* was always present but never abundant. Together, the plant macroremains and charcoal compositions per site revealed similar past communities to those that currently dominate the sandy areas, that is, a *P. banksiana* forest with scattered *P. mariana*. The fungus, *C. geophilum*, was almost always recorded. It characterizes water stress communities as seen currently in the study sites. All other species of trees, shrubs or herbs fluctuated stochastically without a change in plant species richness for at least 6400 years. Objectively, the charcoal layers report a history of fire, but the numbers of paleosoils (layers) do not represent fire abundance at each site. Indeed, the Lunette profile recorded at least nine layers within 5000 years, which would represent a mean apparent fire frequency of 0.0018 fire ky^−1^, that is, a mean fire interval (MFI) of 560 years. At Villemontel, the 17 charred layers over 6400 years represented a fire frequency of 0.0027 fire ky^−1^, that is, a MFI of 370 years. The charred layers are definitely fewer in number than the expected number of fires in such a type of ecosystem in the area, which today burn with fire return intervals of about 110 years in average (based on mean stand age, Bergeron and others 2004) and less than 100 years further north (Le Goff and Sirois 2004; Héon and others 2014). Further southwest, in the northwestern Wisconsin plains under continental climate, it was found MFI of 110–140 years using lacustrine charcoal over the past 2500 years around lakes with *P. banksiana* forest on sandy soils (Lynch and others 2014).

Here, both sites presented the same increase in charcoal layers per time unit during the last 2500 years. This pattern suggests that the number of fires has increased during the late Holocene. Interestingly, this observation matches a higher fire occurrence reported in similar dunes/sandy areas during the last 2000 years in a forest–tundra region situated about 500 km further north (Filion and others 1991; Desponts and Payette 1993; Arseneault and Sirois 2004). A fire reconstruction based on sedimentary charcoal in Lake Pas-de-Fond, situated on an esker about 30 km west of the present study sites, also indicated an increase in fire frequency over the last 2200 years (Carcaillet and others 2001), and more generally in the boreal shield since 3000–2500 years although the fire cycle has tremendously increased since the middle nineteenth century (Le Goff and others 2007; Hennebelle and others 2018). Taken together, this evidence suggests local fire activity coherent with a regional pattern in *P. banksiana* dry areas that has become more active after 2500 cal BP.

### A Long-Term Steady State

The forest appears to have been rather stable for 5000 years, as indicated by plant richness and composition, and sclerote abundances of the ectomycorrhizal fungus *C. geophilum* (Figure 6). According to the relationship between ecosystem and community composition (de Mazancourt and others 2013), this biotic stability would illustrate the stability of the ecosystem despite likely change in fire occurrences. Richness stability has already been reported regionally, based on pollen data from lake sediments in this area (Carcaillet and others 2010) and more widely in the eastern Canadian shield (Blarquez and others 2014). A permanent but stochastic abundance of sclerotes has also been recorded in similar macroremain assemblages from a dune site 500 km to the north (Figure S2 redrawn from Lacroix and others 2011, to match the present Figure 6). However, the stochastic distribution of sclerote abundances hinders any correlation with other proxies, including fire history or *P. banksiana* charcoal and macroremains.

The only significant change was a multi-millennial and monotonic decrease in *P. banksiana*, indicated by the percentages represented in charcoal and macroremains at both sites (Figure 6B, C). The change represents a decrease by 40% during the last 4000 years, that is, 1% of decrease per century, which is a slight rate of change. This pattern supports a regional trend driven by local mechanisms. However, this trend does not match the timing of change in the frequency of charred layers around 2500 cal BP, indicating that the slight and monotonic decrease in *P. banksiana* abundance was not directly linked to a change in fire frequency or to a single fire exceptional in severity. An indirect linkage cannot be ruled out and could be the result of accumulated fires on populations of *P. banksiana* causing a demographic effect related to the length of fire intervals, which were probably locally longer around 5000 cal BP matching regional fire frequency reconstructions (Carcaillet and others 2010; Hennebelle and others 2018) or regional biomass burning activity in the eastern Canadian shield boreal forest (Bremond and others 2010). *P. banksiana* is a short-living species (Desponts and Payette 1992), and potentially more competitive species (*P. mariana*, *P. glauca*, *Abies balsamea*) have better longevity and better regeneration rates with time since last fire (Bergeron and Dansereau 1993; Le Goff and Sirois 2004; Smirnova and others 2008). However, no taxa appeared to be clearly favored by the decrease in *P. banksiana*, and no clear pattern of pine substitution was obvious (Figure 6B, C). Although not significant, percentages of ericaceous charcoal increased more at Lunette than at Villemontel; however, the macroremains showed no such pattern. This percentage increase could be the mathematical result of a decrease in pine percentage without any underlying biological mechanism, that is, a bias of data representation typical of percentage data. Indeed, the macroremains show higher numbers of *P. mariana* fragments, especially at Lunette (Figure 4B), but no distribution trend could be modeled (Figure 6C), and this was not supported by the charcoal data (Fig. 4A). A biological explanation for the multi-millennial decrease in *P. banksiana* could be a reduction in pine biomass or density linked to repeated crown fires without an effect on other canopy species (Arseneault 2001; Pacé and others 2019b). This mechanism probably involves a ground mat of lichens that reduces the regeneration and growth of pine seedlings due to different soil processes, for example, nutrient inhibition, depleted mycorrhizal activity or antimicrobial reactions (Pacé and others 2019a, b). The high charcoal occurrence (65–67%) of the dwarf ericaceous *A. uva*-*ursi* strengthens this hypothesis of soil processes limiting pine recruitment. Indeed, leaf leachate of *A. uva*-*ursi* generates high concentrations of phenolic compounds that reduce nitrification rates and thus inhibit pine regeneration (MacKenzie and DeLuca 2006). The observed progressive decrease in *P. banksiana* (1% 100 y^−1^) could just result from a slight and monotonic decrease in tree productivity and an increase in ground lichen cover and associated soil functionality (Pacé and others 2019a). Unfortunately, lichen cover cannot be recorded by current paleoecological methods. Interestingly, pollen-based studies from lakes surrounded by jack pine generally show a slight increase in *P. banksiana* percentage since less than 3000 years in the area (Liu 1990; Garralla and Gajewski 1992; Carcaillet and others 2001), in the north (Richard 1979; Gajewski and others 1996) or in the southwest (for example, Brubaker 1975; Genries and others 2012; Lynch and others 2014). However, when these data are expressed in influx, the pattern can be different, revealing a decreasing trend during the last millennia (Carcaillet and others 2010) or no change (Liu 1990) suggesting that some long-term processes occurred on jack pine woodlands depending on sites. Generally, these lacustrine-based studies did not include plant remains, and when this proxy was used (Liu 1990), it is interesting to note a general absence of remains of *P. banksiana*.

Contrary to boreal mountains of southern Quebec that experienced a switch under the effect of disturbances during the Holocene from closed and tree-diversified forest to an alternative steady ecosystem characterized by open taiga woodland dominated by *Picea* and ground lichens (Jasinski and Payette 2005), our study context indicates continuous record of *Pinus* without plant diversity erosion nor obvious transformation of the system. In summary, the ecosystem state appears to be steady in terms of plant richness and composition whatever the fire conditions, although the modeled distribution of *P. banksiana* percentage reveals a gentle and monotonic decrease over the last 4000 years. Fire regimes cannot be accurately reconstructed based on the type of data used here; however, the system autocorrelated well in time despite frequent disturbances and water stress, supporting the idea of high resistance (inertia of the system after a disturbance or a stressor effect; Connell and Sousa 1983) or high resilience (recovery rate that is a function of resistance and the time required to return to the initial state after disturbance or a stressor; Holling 1973). Resistance and resilience are two different concepts in disturbance ecology, but they are linked (Walker and others 2004; Nimmo and others 2015); resistance is an intrinsic component of resilience (Figure S1).

### Resilient or Resistant?

The ecosystem was steady over time according to the following facts: (1) *P. banksiana* has dominated the whole series despite a slight decrease and thus figures its contribution to total primary productivity of the ecosystem (‘mass-ratio hypothesis’; Grime 1998), (2) the species richness was stable despite the stochastic record, (3) the fungus *C. geophilum* occurred permanently despite a high variance in sclerote numbers, and (4) a likely increase in fire occurrences after 2500 cal BP not correlated with changes in plant assemblages. It has thus maintained an elevated temporal autocorrelation (similarity between two consecutive observations; permanent dominance of *P. banksiana*) whatever disturbances or other climate changes that, in the first instance, could support hypotheses of high resistance (large inertia) but also of high resilience thanks to strong recovery rate. This means that, whatever the resistance of the system, strong or weak, the recovery rate was high and brief (Figure 1A, B) and the variance of the system was low because never alternative species dominated instead of *P. banksiana*, thus rejecting the scenario of a large basin of attraction (Figure 1C). A recovery time longer than the fire intervals (*R*_*λ*_ = d*t*_*φ*_:d*t*_*λ*_ <1) altering the ecosystem state is probably not realistic (Figure 1D), because no intermediate species were detected in the record (Ericaceae, *Betula*, *Salix*, etc.), and the pine record was continuous. Further, if very long delayed recovery time occurred (MFI>200 y), the system would have switched to a dominance of *P. mariana*, whatever the fire severity (Le Goff and Sirois 2004; Smirnova and others 2008), a species little recorded. If a very short recovery time occurred due to too frequent fire (MFI<50 y), the system would have switched to a non-forest community of shrubs, lichens and herbs (Hart and others 2018); shrubs and herbs are taxa well recorded in plant macroremains or charcoal but stochastic and never abundant (Figure 4). Even if fire frequencies had varied during the past 6000 years, they were probably within the range of intervals observed during the last two centuries (Le Goff and others 2007; Héon and others 2014). This situation is guaranteed, at least, by the short recovery time of pine canopy, and has led to a strong resilience of this ecosystem whatever its resistance.

The possibility that the ecosystem resistance was weak (Figure 1B) or strong (Figure 1A) has to be considered as a theoretical hypothesis. The weak resistance associated with a fire-prone community assumes that the resilience potential of the ecosystem thanks to a rapid recovery time associated with high autocorrelation offsets the temporary loss of functionality as a result of disturbance. In contrast, the strong resistance assumes that, whatever the environmental changes (chronic, as with climate change or soil maturation, or transitory, as with disturbance), the functionality of the system does not change, which includes the plant community that is manifested by the complex functioning of the ecosystem (analogy with a response trait for an organism). Today, dry sandy areas in Canadian boreal forests mainly host *P. banksiana* and *P. mariana* in the canopy and ericaceous or ground lichens in the understory. *Pinus* abundance is controlled by fires (Johnson 1992) but is generally excluded by intervals of stand-replacing fire longer than 200 years, while *P. mariana* is excluded by intervals shorter than 60 years (Desponts and Payette 1992; Le Goff and Sirois 2004; Splawinski and others 2019). Temporary modification of the canopy from *P. banksiana*-dominant to *Picea* dominant during the last 6000 years would thus show a loss of autocorrelation over a period of 500 to 1000 years. Such loss would result from the elongation of fire-free intervals altering the understory and biogeochemical properties, notably an increase in residence time of organic matter, which potentially favors the regeneration of *P. mariana* by layering (Laberge and others 2001; Figure 2) and alters ectomycorrhizal functionalities (Pacé and others 2019a). The only trend associated with the gentle decrease in pine is the slight augmentation of Ericaceae recorded in the charcoal assemblages of Lunette (Figure 6B), which might be linked to more severe fires stimulating ericaceous species instead of *Cladina*-type lichens more adapted to less severe fires (Arseneault 2001; Pinno and Errington 2016). However, the assemblages from Villemontel do not show such a pattern, suggesting that fire severity finally did not change significantly over time. Furthermore, *Picea* was never dominant at either Lunette or Villemontel, suggesting that the fire intervals remained short (high fire frequency) and the fires were severe enough to hinder *Picea* regeneration by seed or by layering (Walker and others 2018). Based on our paleobotanical data, the scenario of elongation of fire-free intervals is thus not realistic and was certainly not met during the last 6000 years. Finally, the decrease in pine abundance revealed by both macroremains and charcoal at both sites may illustrate a sustained inhibition of pine regeneration that could be the result of soil processes linked to the ground mat of lichens (Pacé and others 2019a) or ericaceous species (Nilsson and Wardle 2005). The high regeneration of pine in a high-frequency fire regime explains the steady state (autocorrelation), and the partial inhibition of pine regeneration or growth (Pacé and others 2019a) explains the slight trend observed over millennia, that is, a decrease in pine percentage. The system would thus tend to be resistant.

Interestingly, in regional sites situated 500 km further north, *P. banksiana* and *P. mariana* were either codominant for 4800 years (Arseneault and Sirois 2004), where *Picea* abundance decreased in favor of *P. banksiana* less than 3000 years ago, suggesting a fire-linked ecosystem functioning (Desponts and Payette 1993; Lacroix and others 2011). The pattern reported in Lacroix and others (2011; Figure S2) seems rather important, triggering the rarefaction of *Picea* and eventually the creation of an alternative state dominated by pine. Contrary to these observations at sites close to the forest–tundra transition, *ca*. 500 km north of the current study sites, our present observations from the core of the boreal forest indicate that a steady state dominated by pine was initially established 6400 years ago. Because over millennia the canopy species (*P. banksiana*) is also the plant that recruits abundantly shortly after a fire, the ecosystem appears to be at least as resistant as it is resilient to all accumulated environmental changes.

Finally, the continuous but slight decrease in abundance of the dominant tree (Figure 6) would figure the slight decrease in ecosystem functionality (sensu ‘mass-ratio hypothesis’; Grime 1998) despite indicators suggesting a metastable system over time. However, this slight decrease within a general steady state is associated with a disturbance regime (here underestimated by analogy with modern context of the eastern Canadian boreal forest, for example, Le Goff and others 2007; Héon and others 2014), which questions the hypothesis that a forest ecosystem needs disturbance to secure its high productivity by stimulating microbial activities and biogeochemical processes (Wardle and others 2004; Petzler and others 2010). The fire frequency during the last 4000 years was maybe not high enough to stimulate processes associated with bacterial flora instead of fungi flora, which should promote an increase in forest productivity (Wardle and others 2004). Or, fires were too frequent preventing the growth of other tree species (*Picea*, *Betula*, *Alnus*), which would have effects increase the ecosystem productivity notably by complexing the trophic network, reducing the strength of demographic stochasticity and enhancing compensatory processes (de Mazancourt and others 2013). However, fires were maybe too severe to allow for a good regeneration and soil functionality. Whatever the theory and generalization, here the chronology shows a general steady system frequently disturbed by fires with a slight decrease in the only dominant species suggesting a slight diminution of the productivity over time. This paleoecological analysis matches a modern large-scale spatial analysis indicating a high resilience of *P. banksiana* forest with current regime of fire frequency (Hart and others 2018). Ecosystem dominated by *Pinus banksiana* on dry soils is thus a multi-millennial steady state and resilient ecosystem.

## Conclusion

Dry sandy areas of the east Canadian boreal forest have been stable and covered by *P. banksiana* for at least 6000 years despite harsh conditions resulting from frequent burnings and a substratum subjected to water stress. This is a general context theoretically reducing the role of competition as driver, and thus selecting species adapted to stress and disturbances (Grime 1977). Tree-cover and species diversity remained relatively stable, despite the presence of other species adapted to stress or disturbances, for example, *P. mariana* and Ericaceae. The only transient modification has been a multi-millennial, slight decrease in *P. banksiana* abundance without any clear substitution by another species or species group. The system appears to be resistant and mostly resilient, leading to a high autocorrelation without rapid change in plant communities and richness, which represents an ecosystem characterized by a steady state. However, because the ecosystem resilience is not total, the ecosystem has changed subtly over millennia, recording a slight reduction in *P. banksiana* abundance with a rate of 1% per century.

## Electronic supplementary material

Below is the link to the electronic supplementary material.

Supplementary material 1 (PDF 1638 kb)
