# Supplementary material for: Long-Term Steady-State Dry Boreal Forest in the Face of Disturbance
Source: Ecosystems. 2019 Oct 30;23(5):1075–92. doi: 10.1007/s10021-019-00455-w (PMC7410099; doi:10.1007/s10021-019-00455-w)
Supplement: Supplementary file 1 — Supplementary material 1 (PDF 1638 kb) [file 10021_2019_455_MOESM1_ESM.pdf]

Long-term steady-state dry boreal forest in the face of disturbance

Christopher Carcaillet[1,2] *, Mireille Desponts[3], Vincent Robin[4], Yves Bergeron[5]

1) Laboratory for Ecology of Natural and Anthropised Hydrosystems (UMR 5023 CNRS ENTPE), Université Claude Bernard-Lyon, F-69622 Villeurbanne, France

2) Paris Sciences & Lettres Université (PSL), École Pratique des Hautes Études (EPHE), Paris, France

3) Ministère des Forêts, de la Faune et des Parcs, Gouvernement du Québec, Direction de la recherche forestière, 2700 rue Einstein, Québec, Québec G1P 3W8, Canada

4) Interdisciplinary Laboratory for Continental Environments (LIEC), University of Lorraine, CNRS, Campus Bridoux, Rue du Général Delestraint, F-57070 Metz, France

5) Institut de recherche sur les forêts, Université du Québec en Abitibi-Témiscamingue, 445 boulevard de l'Université Rouyn-Noranda, Québec J9X 5E4, Canada

* Correspondence: 00 33 609 93 16 94 | christopher.carcaillet@ephe.psl.eu

S1. Resistance and resilience (generalities)

Resistance (Connell and Sousa 1983) and resilience (Holling 1973) are two different concepts in disturbance ecology, but they are linked (Walker and others 2004; Nimmo and others 2015). Resistance (*h*) corresponds to the inertia of an ecological system facing a disturbance, or the capacity of the system to absorb a disturbance (Fig. S1). Resilience (λ) is the recovery rate, which is a function of resistance and the time required (dt_λ) to return to the initial steady state (Fig. S1). The recovery of the steady state can be assessed by autocorrelation ($Rx(\tau)$), which measures the similarity between the state before and after a disturbance (Dakos and others 2015). Explicitly, the assessment of $Rx(\tau)$ requires a component rarely described in the ecology of aeolian dunes: the ecosystem state before the disturbance (Nimmo and others 2015). Implicitly, paleoecology or long-term studies provide an assessment of the original state, steady or unstable (Dakos and others 2008). In the long term, repeated disturbances or changes in fire severity can alter ecosystem resilience and contribute to change either through resistance loss (Fig. S1f) or by altering the recovery time as a result of altered functionality (Fig. S1e). The ecosystem structure (biomass, diversity) can also change if the time between disturbances does not adjust with the recovery time (Fig. S1d).

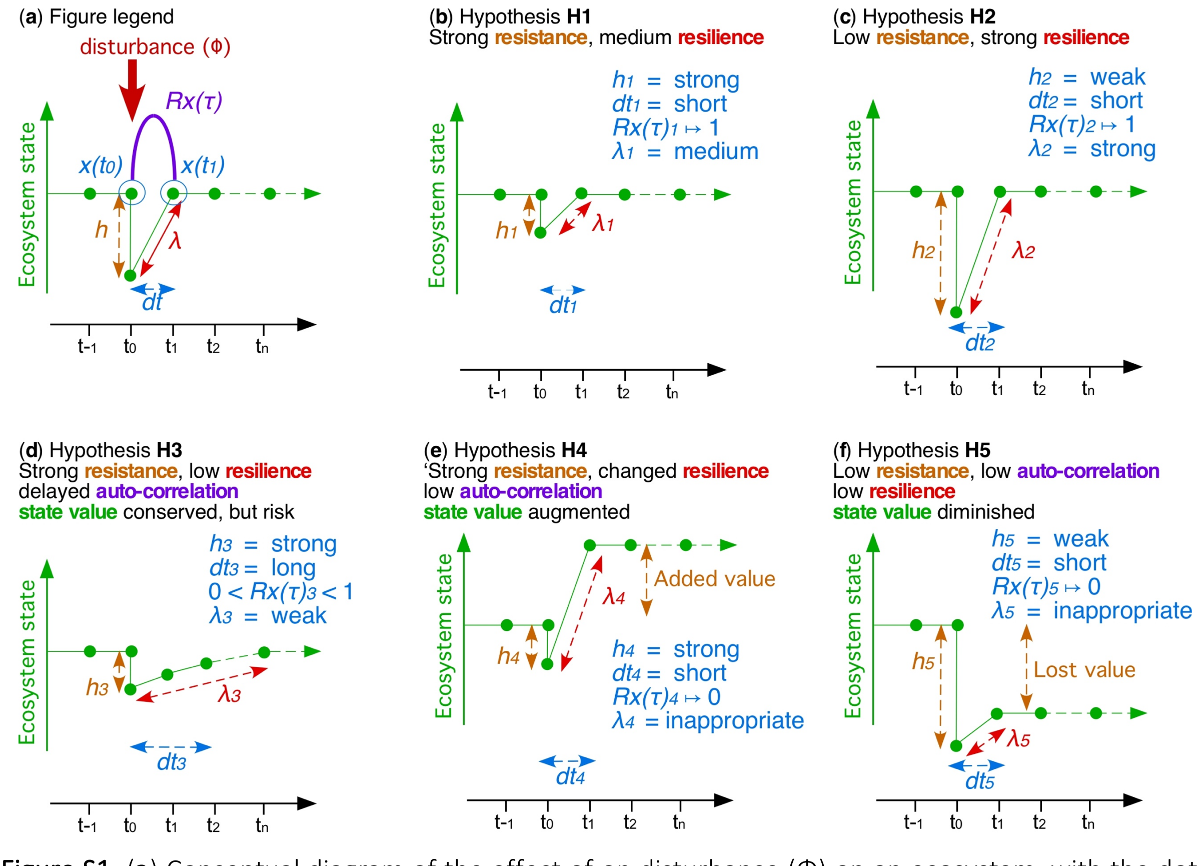

Figure S1. (**a**) Conceptual diagram of the effect of on disturbance (Φ) on an ecosystem, with the dots representing ecosystem states at time t_n, dt_λ the recovery time, *h* the resistance or the difference in the ecosystem after the disturbance, λ the resilience or recovery rate as $\lambda = f(\Phi, h, dt_\lambda)$, and $Rx(\tau)$ the autocorrelation of the ecosystem before $x(t_0)$ and after $x(t_1)$ the disturbance, with $Rx(\tau)$ ranging from 0 to 1, where 0 is no correlation and 1 the maximum. (**b**) Strong resistance, whatever the resilience value, which conserves the functionality of the ecosystem, as revealed by a high autocorrelation $Rx(\tau) = 1$. (**c**) Weak resistance but strong resilience because of a quick recovery time. (**d**) Strong resistance but delayed recovery time without loss of a steady state; this situation exposes the system to risk if a disturbance occurs before $Rx(\tau) = 1$, which can occur at $t_n > t_1$. (**e**,**f**) Resilience loss resulting in a low autocorrelation and a change in ecosystem functionality or state, either augmented (**e**) or diminished (**f**).

S1.1 Reference

Connell JH, Sousa WP. 1983. On the evidence needed to judge ecological stability or persistence. American Naturalist, 121:789–824

Dakos V, Scheffer M, van Nes EH, Brovkin V, Petoukhov V, Held H. 2008. Slowing down as an early warning signal for abrupt climate change. Proceedings of the National Academy of Sciences of the USA, 105:14308–14312.

Dakos V, Carpenter SR, van Nes EH, Scheffer M. 2015. Resilience indicators: prospects and limitations for early warnings of regime shifts. Philosophical Transactions of the Royal Society B, 370:20130263.

Holling S. 1973. Resilience and stability of ecological systems. Annual Review of Ecological Systems, 4:1–23.

Nimmo DG, Rally RM, Cunningham SC, Haslem A, Bennet AF. 2015 Vive la résistance: reviving resistance for 21st century conservation. Trends in Ecology and Evolution, 30:516-523

Walker B, Holling CS, Carpenter SR, Kinzig A. 2004. Resilience, adaptability and transformability in social–ecological systems. Ecology and Society, 9:5. www.ecologyandsociety.org/vol9/iss2/art5/

S2. Taphonomy of charcoal and other macroremains (plants, fungi)

S2.1. Charcoal taphonomy

The taphonomy of charcoal is probably not very different between species after burning, although we can suspect differences of resistance to fragmentation in soil, notably weaknesses for wood charcoal of *Populus* and *Salix* due to their very thin wood cell wall (Carcaillet 1996). In the absence of proof of inter-specific processes, soil charcoal taphonomy are considered homogeneous, even preserved in different boreal soil types, organic or mineral (De Lafontaine and Asselin 2011). However, homogeneous processes include homogeneous degradation with time. If the biochemical oxidation occurred mainly during month and years following the charcoal production mainly in response to charring conditions (Zimmerman and others 2012; Naisse and others 2013), it is obvious based on observations, experimentations or rational (theoretical thinking) that the charcoal stock is not stable through time due to several processes including cryofraction and rhizofraction (Carcaillet 2001), and simply by secondary consumption of charcoal by fires in regions without burying of fragments by bioturbation (Pingree and others 2012). Indeed, when charcoals are not protected in the soil matrix that is the case in boreal ecosystem where burying by bioturbation is an exception, fragments remain in the litter horizon or at the interface between soil and organic horizon until a severe fire will consume all organic matters including charcoal, and transform them in ashes. These transformation processes explain certain discrepancies between soil charcoal chronology and sedimentary charcoal chronologies, notably when first millennia of the Holocene are compared, resulting from higher probability of charcoal decay with time in boreal soils (Ohlson and others 2013; Couillard and others 2019). Charcoal contained in paleosoils buried under sand accumulations in dune fields are naturally protected from this cumulative effect of sever fires, which decay or mineralize charcoal by burning, although biochemical decay on the very long-time (>10 000 yrs) remains an interrogation. Charcoal from dune layers allow thus to report very old fire history in a similar pattern as charcoal from lacustrine sediment (cf. Carcaillet and others 2007 *vs* Matthews and Seppälä 2014).

This means that the proportion of species should be conservative through time and not altered by soil mechanisms in boreal ecosystems. The main source of differences would be during the burning process, due to (i) the structure of the woody community and (ii) the species flammability. These factors could result little differences in terms of species within assemblages; indeed, if fires are poorly severe (surface fires), then charcoal assemblages should be composed of ground biomass chiefly, i.e. shrubs and ground woody debris (Bégin and Maguerie 2002), while if fire are highly severe, species from the canopy should also contribute to the assemblage. However, the ground woody debris are generally similar to the tree canopy, unless the burned community was experiencing a change from a past dominant species abundant in woody debris and the new dominant species not yet present in the

woody debris. In jack pine forests, fires are usually highly or moderately severe (Smirnova and others 2008). The only aspect that varies is the burning rate of species, a process that we have already analyzed in the European Alps with species having great differences in ignition and consumption (Fréjaville and others 2013). If we consider for the Canadian boreal species the values measured on the European species with similar phylogeny, then *Picea mariana* and *Pinus banksiana* would both have the same flammability components, but they would be less flammable than *Betula* and other broadleaf species. Explicitly, broadleaf trees in boreo-mountain forests (i.e. *Betula*, *Salix*, *Populus*, *Alnus*) are expected to produce more charcoal than needleleafs, everything being equal (biomass). Bark thickness and wood density explain half of combustibility variance everything being equal (tree diameter, tree age). Unfortunately, such type of studies seems missing in the boreal, and more generally on shrubs, e.g., Ericaceae and *Juniperus*. However, this plant trait (wood flammability) is conservative, meaning that a species that burns well will every time burn well. Consequently, because the analysis is based on percentage of species in charcoal assemblages all coming from the same type of archive (dune layers), we assume that temporal variabilities in taphonomy has low chance to alter through time the present assemblages.

S2.2. Macroremains taphonomy (plants, fungi, lichens)

Surprisingly, while plant macroremains have a very long history of usage in scientific research, this is more complex to provide strong arguments on their taphonomy in natural archives, because most taphonomical studies are concerned by archaeological contexts (Cappers 1995). We can indeed consider different processes of preservation of plant remains according to species and their residence time in the soil litter. Indeed, ligneous materials (e.g., wood, cone) have more chance to be preserved compared to cellulosic materials that characterize herbs and part of woody plants notably needles and leaves. Furthermore, soft (high % of water) and unprotected seeds (e.g. *Pinus banksiana*, *Picea sp.*, *Abies balsamea*, Poaceae, Salicaceae, etc.) are generally rapidly consumed in soils by animals or fungi if they did not germinate immediately. On the contrary, some dried of protected seeds (e.g. *Prunus* sp., *Arctostaphyllos uva-ursi*, *Vaccinium* sp.) can be preserved during years to decades in soils making these seeds good candidate for conservation if the soil is buried by sand. Unfortunately, we don’t know any studies on the taphonomy of plant remains in soil/dunes. We thus assume that the conservation processes are stable trough time, whatever the differences between species, meaning that assemblages are comparable each others.

The only fact is that plant macroremains are more likely to deform plant community image than wood charcoal due to species-related process of preservation evoked above whatever the guild of plant. Wood charcoal reports only the guild of the woody plants (trees, shrub), but not the herb layers composed of celluloses that generally trigger a total consumption of plant material during the burning.

Finally, sclerotes of *Cenococcum geophilum* are propagules that are dormant for times in soils during period of stress for its ectomycorrhizal host, which can be a tree, a fern or a herb, i.e. more than 200 species known as potential host (Molina and Trappe 1982). This biological structure is very resistant to degradation and remains in soils for years (Trappe 1969), and can be very abundant until hundreds of kg in boreal forest soils (Dahlberg 1997). We do not know any taphonomic studies on *C. geophilum*, while sclerotia are well known to paleoecologists (van Geel 1978; Ali and others 2008; Scott and others 2010)

Very important boreal species like lichens or eventually mosses, might have expanded through time, playing a rising role in the functioning of the ecosystem. Unfortunately, lichens and mosses are not preserved (or exceptionally). Practically, this fact thus limits somehow the interpretation specific to ecosystems where ground lichens are very important functional species. Notably, the observed decrease of *Pinus banksiana* since 4000 years is not compensated by any other woody species or by herbaceous plant. This could result from the increase abundance and cover of ground lichen, and all associated processes on growing inhibition and seedling (Pacé and others 2019).

S2.3. Reference

Ali AA, Asselin H, Larouche AC, Bergeron Y, Carcaillet C, Richard PJH. 2008. Changes in fire regime explain the Holocene rise and fall of *Abies balsamea* in the coniferous forests of western Québec, Canada. The Holocene, 18:693-703.

Bégin Y, Marguerie D. 2002. Characterization of tree macroremains production in a recently burned conifer forest in northern Québec, Canada. Plant Ecology, 159:143-152.

Cappers RTJ. 1995. A palaeoecological model for the interpretation of wild plant species. Vegetation History and Archaeobotany, 4:249-257.

Carcaillet C. 1996. Évolution de l'organisation spatiale des communautés végétales d'altitude depuis 7000 ans BP dans la vallée de la Maurienne (Alpes de Savoie, France): une analyse pédoanthracologique. Ph.D. Thesis, Université Aix-Marseille 3, France.

Carcaillet C. (2001) Soil particles reworking evidences by AMS ^{14}C dating of charcoal. Comptes Rendus de l'Académie des Sciences Paris, Série Sciences de la Terre et des Planètes 332, 21-28

Carcaillet C, Bergman I, Delorme S, Hörnberg G, Zackrisson O. 2007. Long-term fire frequency not linked to prehistoric occupations in northern Swedish boreal forest. Ecology, 88:465-477.

Couillard PL, Tremblay J, Lavoi M, Payette S. 2019. Comparative methods for reconstructing fire histories at the stand scale using charcoal records in peat and mineral soils. Forest Ecology and Management 433:376-385.

Dahlberg A. 1997. Population ecology of *Suillus variegatus* in old Swedish Scots pine forests. Mycological Research, 101: 47-54.

De Lafontaine G, Asselin H. 2011. Soil charcoal stability over the Holocene across boreal northeastern North America. Quaternary Research, 76:196-200.

Frejaville T, Carcaillet C, Curt T. 2013 Calibration of charcoal production from trees biomass for soil charcoal analyses in subalpine ecosystems. Quaternary International, 289:16-23.

Matthews JA, Seppälä M. 2014. Holocene environmental change in subarctic aeolian dune fields: The chronology of sand dune re-activation events in relation to forest fires, palaeosol development and climatic variations in Finnish Lapland. The Holocene, 24:149-164.

Molina R, Trappe JM. 1982. Patterns of ectomycorrhizal host specificity and potential among pacific Northwest conifers and fungi. Forest Science, 28:423-458.

Naisse C., Alexis M., Plante A., Wiedner K., Glaser B., Pozzi A., Carcaillet C., Criscuoli I., Rumpel C. (2013) Can biochar and hydrochar stability be assessed with chemical methods? Organic Geochemistry 60, 40-44

Ohlson M, Kasin I, Wist AN, Bjune AE. 2013. Size and spatial structure of the soil and lacustrine charcoal pool across a boreal forest watershed. Quaternary Research, 80:417-424.

Pacé M, Fenton NJ, Paré D, Stefani FOP, Massicotte HB, Tackaberry LE, Bergeron Y. 2019. Lichens contribute to open woodland stability in the boreal forest through detrimental effects on pine growth and root ectomycorrhizal development. Ecosystems, 22:289-201.

Pingree MRA, Homann PS, Morrissette B, Darbyshire R. 2012. Long and short-term effects of fire on soil charcoal of conifer forest in Southwest Oregon. Forests 3, 353–369.

Scott AC, Pinter N, Collinson ME, Hardiman M, Anderson RS, Brain APR, Smith SY, Marone F, Stampanoni M. 2010. Fungus, not comet or catastrophe, accounts for carbonaceous spherules in the Younger Dryas "impact layer". Geophysical Research Letters, 37:L14302.

Smirnova E, Bergeron Y, Brais S. 2008. Influence of fire intensity on structure and composition of jack pine stands in the boreal forest of Quebec: live trees, understory vegetation and dead wood dynamics. Forest Ecology and Management, 255:2916–2927.

Trappe JM. 1969. Studies on *Cenococcum graniforme* I. An efficient method for isolation from sclerotia. Canadian Journal of Botany, 47:1389-1390.

van Geel B. 1978. A palaeoecological study of Holocene peat bog sections in Germany and the Netherlands. Review of Palaeobotany and Palynology, 25: 1-120.

Zimmermann M, Bird MI, Wurster C, Saiz G, Goodrick I, et al. 2012. Rapid degradation of pyrogenic carbon. Global Change Biology, 18: 3306–3316.

Table S1. List of vascular plant remains (X) and charcoal (C) in the dune paleosoils of Villemontel. Charcoal taxa like Larix/Picea or unidentified conifer or broadleaf shrub or tree have not been included in the table.

	Depth (cm)																	Occ. (%)
Taxa	9.5	18.0	38.5	79.5	95.5	103.5	116.5	126.5	140.5	149.5	167.5	174.5	186.5	190.5	202.5	206.0	221.5	
Larix laricina				X C		C											X	18
Picea marianna		X		X	X	X	X		X	X	X	X	X				X C	65
Pinus banksiana	X C	X C	X C	X C	X C	X C	C	C	X C	X C	X C	X C	X C	C	C	X C	X C	100
Aralia hispida	X	X			X			X	X	X	X	X					X	53
Arctostaphylos uva-ursi		X	X	X	X			X		X	X	X	X	X		X		65
Cornus canadensis			X								X						X	18
Diervilla lonicera				X	X							X				X	X	29
Empetrum		C		C	C		C				C	C				C		41
Hudsonia tomentosa	X	X		X								X	X					29
Prunus pensylvanica		X	X							X						X		24
Vaccinium angustifolia	X	X																12
Vaccinium vitis-idaea		X																6
Viola sp.																X		6
Carex sp. 1 (2-sides seed)								X		X				X			X	24
Carex sp. 2 (3-sides seed)	X	X			X	X		X	X	X	X		X	X	X		X	71
cf. Panicum sp.			X															6
Scirpus sp.																X		6
Comptonia peregrina	X																	6
Equisetum sp.																	X	6
Lycopodium tristachyum	X																	6
Richness (*S*)	7	10	5	7	7	4	3	5	4	7	7	7	5	4	2	7	9	

Table S2. List of plant remains (X) and charcoal (C) in the dune paleosoils of Lunette. Taxa like Larix/Picea or unidentified conifer or broadleaf shrub or tree (charcoal) have not been included in the table. The Richness (*S*) excludes the Polytrichum (bryophytes).

	Depth (cm)									Occurrence (%)
Taxa	12.0	37.5	59.0	78.5	88.5	158.0	172.5	192.5	208.5	
Abies balsamea						X		C		11
Larix laricina									X	11
Pinus banksiana	X C	X C	X C	X C	X C	X C	X C	X C	X C	100
Picea mariana	X	X	X		X	X	X	X		78
Arctostaphylos uva-ursi	X		X	X		X	X	X		67
Aralia hispida	X		X	X						33
Cf. Empetrum	C	C		C		C	C			56
Hudsonia tomentosa	X	X	X	X	X	X	X			78
Cf. Ledum	C	C	C	C						44
Prunus pensylvanica					X					11
Carex sp. 1 (2-sides seed)								X		11
Carex sp. 2 (3 sides seed)									X	11
Poaceae			X					X		22
Equisetum sp.								X		11
Richness (*S*)	7	5	7	6	4	6	5	8	3	

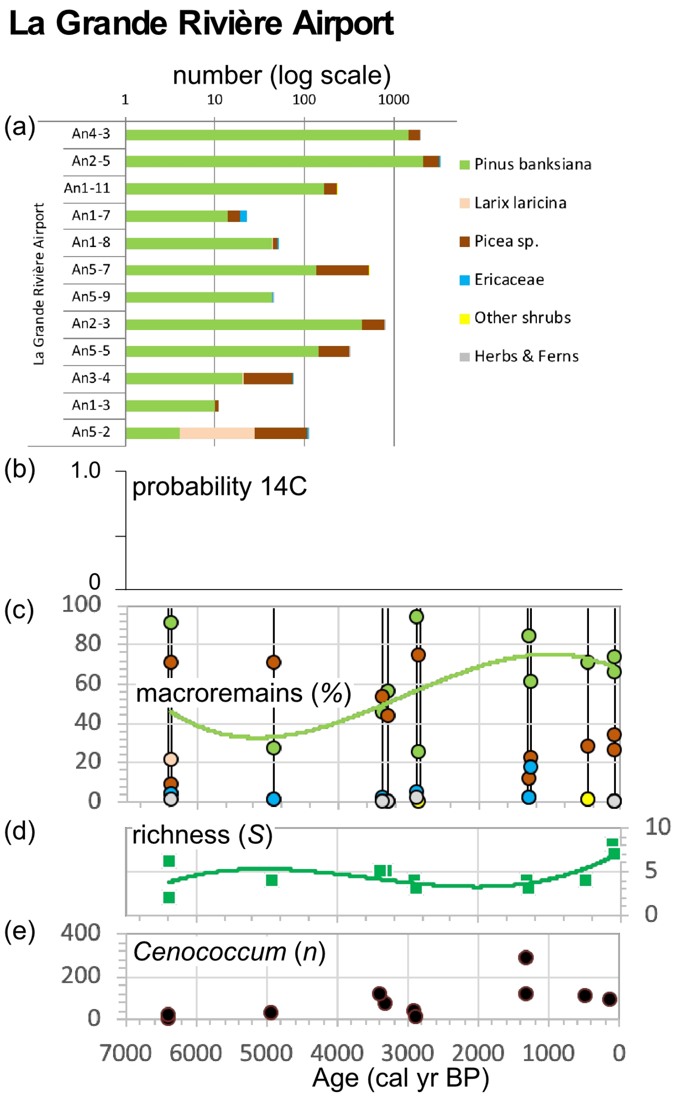

Figure S2. Plant and biodiversity trajectories at La Grande Rivière Airport (adapted from Lacroix et al. 2011), inferred from assemblages of charcoal and macroremains from paleosoils in western Quebec, Canada (53°37'N; 77°41'W). (**a**) Plant macroremains (number per cm, log scale) plotted against depth, from the youngest layer (An4-3) to the oldest (An5-2); layers are stratified; each layer corresponds to a paleosoil containing remains from one or several fire intervals. (**b**) Probability sums of ^{14}C dating. (**c**) Plant macroremain frequency (%) and temporal distribution of *P. banksiana* based on third-order polynomials; the vertical bars correspond to ages of burned paleosoils simulated based on age~depth models; same legend as in the figure 5 of the main text (**d**) Vascular plant richness based on macroremains; temporal distribution based on third-order polynomials (mean richness ±SE = 4.6 ±0.6). (**e**) Sclerote numbers of *Cenococcum geophilum*, an ectomycorrhizal fungus.

Unfortunately, in the study of Lacroix et al. (2011), charcoal fragments were not identified, making this study partially comparable to the study at Lunette and Villemontel reported in the main text.

Reference (Fig. S2)

Lacroix C, Lavoie M, Bhiry N. 2011. New macrofossil evidence for early postglacial migration of jack pine (*Pinus banksiana*) in the James Bay region of northwestern Quebec. Ecoscience, 18, 273-278.
